# National eHealth strategy frameworks in Africa: a comprehensive assessment using the WHO-ITU eHealth strategy toolkit and FAIR guidelines

**DOI:** 10.1093/oodh/oqae047

**Published:** 2024-11-20

**Authors:** Isaac Iyinoluwa Olufadewa, Opeyemi Paul Iyiola, Joshua Nnatus, Kehinde Fatola, Ruth Oladele, Toluwase Olufadewa, Miracle Adesina, Joseph Udofia

**Affiliations:** Center for Excellence in Modelling Population Health and Enviroment Risks (CEMPHER), Slum and Rural Health Initiative, 11, Abiodun Akerele Street, Awolowo Avenue, Bodija, Ibadan, Oyo State 200222, Nigeria; Department of Community Medicine, University College Hospital, Queen Elizabeth II Road, Agodi, 200285 Ibadan, Nigeria; Directorate of Medical Administration and Training, Special Project and Mental Health Unit Ministry of Health, Lagos State Government, Alausa, Ikeja, 21007, Lagos, Nigeria; Department of Physiotherapy, Lagos University Teaching Hospital, Ishaga Road, Idi-Araba, Lagos 102215, Lagos, Nigeria; Center for One Health and Zoonotic Diseases (COHAZD), Slum and Rural Health Initiative, Ibadan, Oyo State, Nigeria, 11, Abiodun Akerele Street, Awolowo Avenue, Bodija, 200222, Ibadan, Oyo State, Nigeria; Center for Excellence in Modelling Population Health and Enviroment Risks (CEMPHER), Slum and Rural Health Initiative, 11, Abiodun Akerele Street, Awolowo Avenue, Bodija, Ibadan, Oyo State 200222, Nigeria; Center for One Health and Zoonotic Diseases (COHAZD), Slum and Rural Health Initiative, Ibadan, Oyo State, Nigeria, 11, Abiodun Akerele Street, Awolowo Avenue, Bodija, 200222, Ibadan, Oyo State, Nigeria; Center for Excellence in Modelling Population Health and Enviroment Risks (CEMPHER), Slum and Rural Health Initiative, 11, Abiodun Akerele Street, Awolowo Avenue, Bodija, Ibadan, Oyo State 200222, Nigeria

**Keywords:** digital health, Ehealth national strategy, Africa, policy review, tool, review

## Abstract

Many African nations have developed national eHealth strategies to harness the benefits of digital health solutions. Our study assessed the current state of national eHealth strategies in Africa. A systematic search identified publicly available national eHealth strategy documents published from 2000 to 2023 in Africa. The documents were independently reviewed and scored using the eHealth National Strategy Rating Tool, developed using the WHO-ITU national eHealth strategy toolkit and the Findability, Accessibility, Interoperability and Reusability Guidelines. The eHealth National Strategy Rating Tool covered five domains: (i) eHealth national vision and strategy, (ii) implementation plan, (iii) monitoring and evaluation component, (iv) Findability, Accessibility, Interoperability and Reusability mention and (v) recency of policy development. The study followed a rigorous five-step methodological approach proposed by Arksey & O’Malley (2005). This study found the national eHealth strategies for 34 African countries, with only 16 updated to cover the year 2023 or beyond. Significant variability in the quality and comprehensiveness of national eHealth strategies was observed. Nine countries had ‘strong’, 17 had ‘moderate’, and eight had ‘weak’ eHealth strategies. Critical gaps were identified in the implementation plan, monitoring and evaluation, and the alignment of policies with the Findability, Accessibility, Interoperability and Reusability data principles. Addressing the gaps identified in the development and implementation of national eHealth strategies across Africa by aligning national strategies with global best practices will be crucial for African nations to harness the transformative potential of digital technologies and ensure equitable access to quality healthcare for their populations.

## INTRODUCTION

From telemedicine platforms to mobile health applications, the rapid advancement of digital technologies has profoundly transformed healthcare in Africa and internationally [1, 2]. Furthermore, the emergence of artificial intelligence (AI) and machine learning (ML) technologies and large Language models has enabled the development of predictive analytics tools that can assist healthcare providers in early disease detection and personalized treatment planning [3]. The adoption of these digital health innovations has gained significant momentum across African countries, driven by their potential to address the region’s pressing healthcare challenges and reduce health inequities [4].

eHealth can be described as the delivery of healthcare information and/or services using technology-based applications and infrastructure. It is widely regarded as an innovative and cost-effective medium, and it includes mobile health (mHealth), electronic health records (EHRs), telemedicine and other digitally mediated platforms that address health inequalities [5, 6]. They have been positioned by several national governments as a critical tool for advancing population health and transforming national development [6]. However, the effective integration and scale-up of these technologies require a robust framework to guide their implementation, governance and alignment with national healthcare priorities [6]. Hence, many African nations have developed national eHealth strategies to harness the benefits of digital health and ensure equitable access to quality care for their populations, especially as they face a shortage of skilled health professionals.

In 2012, the World Health Organization (WHO) and the International Telecommunications Union (ITU) published their WHO-ITU national eHealth strategy toolkits, which provide a framework and methods for the development, deployment and evaluation of national eHealth strategies [7]. The WHO-ITU Strategy Toolkits consist of several components such as the framework for action, implementation guidelines, monitoring and evaluation, case studies and best practices covering aspects such as governance, rationale, strategy development, capacity building and resource allocation. Hamilton [8] described it as an effective blueprint for the development of national eHealth strategies. Two years after the development of the WHO-ITU Strategy, the Findable, Accessible, Interoperable and Reusable (FAIR) Guidelines (also known as the FAIR Data Principles) for data management and stewardship to ensure data and metadata are easy to find, access, operate and reuse for humans and machines were developed in 2014 [9]. The FAIR principles help ensure that health data is managed effectively and its implementation can enhance the efficiency of national eHealth systems. However, since 2012, most of the national policies and programs in Africa and globally have not been assessed using these international standards.

A study by Inau *et al.* (2022) explored the regulatory framework in Kenya that enabled the implementation of the FAIR Guidelines in health research [10]. Similarly, studies have been conducted by Kawu *et al.* (2022), Basajja *et al.* (2022) and Taye *et al.* (2022) in Nigeria [11–13], Uganda and Ethiopia, respectively, that critiqued policy documents based on the FAIR Guidelines. Aside from their narrow focus on the FAIR Guidelines, none of these focused on national eHealth or digital health-related strategies. Furthermore, their review was not comprehensive enough to include vital aspects of these policies, such as the eHealth rationale, objectives, implementation plan, resource mobilization plan, monitoring and evaluation and governance. This study fills this huge gulf. After searching various databases, no study has rigorously critiqued national eHealth strategies in African countries. This study is the first to assess national digital health policies across African continents using internationally recognized frameworks and guidelines such as the WHO-ITU National Framework for eHealth Strategy and the FAIR Guideline.

This study is important for several reasons. It examines key elements and governance frameworks in national eHealth policies, allowing researchers and policymakers to identify best practices and areas for improvement. Insights gained can refine these policies, fostering cross-country collaboration and effective digital health integration in Africa. Additionally, the review highlights varying levels of commitment to digital health across countries, revealing gaps and opportunities for targeted interventions. The study also presents a rigorous methodology and tool for future critiques of eHealth strategies, offering valuable insights for healthcare providers, technology companies, investors and international organizations involved in Africa’s digital transformation.

The primary objective of this study was to conduct a rigorous analysis of the national eHealth (or digital health) policies and strategies across African countries using internationally developed standards to understand their current state. Specifically, this study focused on understanding the prioritization of their digital health development agenda, rationale, vision and strategy, implementation framework, governance, monitoring and evaluation components and their alignment with the FAIR Guidelines.

## METHODS AND METHODS

### Overview of methods

A search was conducted for published and publicly available national eHealth or digital health-related strategy documents in all African countries. The review focused on strategy documents that were published from 1 January 2000 to 31 December 2023, as this period marked an increasing focus on the integration of digital health technologies within healthcare systems across the African continent. A rigorous five-step methodological approach proposed by Arksey & O’Malley (2005) was followed to conduct the study. These were (i) the identification of research questions and the development of the eHealth National Strategy Rating Tool, (ii) the identification of relevant documents, (iii) the screening and selection of relevant documents, (iv) data extraction and reporting and (v) data synthesis and reporting. Furthermore, there was an additional step (the sixth step), which was ‘reliability testing’ as an inter-rater reliability test was conducted to further strengthen and improve the reliability of our study.

#### Step 1: identification of research questions and development of the methodological tool

The research team comprising an interdisciplinary team of digital health practitioners, medical doctors, data analysts, technology consultants, physiotherapists, policy analysts, nonprofit organization workers, social scientists and government officers met several times physically and virtually since the conceptualization of the topic in 2022 to identify the research questions and develop the methodological tool.

### Research questions

The research team agreed that the primary research question for this study will be: What is the current state of national eHealth policies and strategies in African countries? Secondary research questions included: How many countries have publicly available eHealth strategies? How many African countries have updated their eHealth or digital health policies? Finally, how did the strategy documents perform on each of the four other domains of the methodological tool developed using evidence-based international eHealth standards (WHO/ITU Strategy and FAIR Guidelines)?

### Development of the eHealth national strategy rating tool

The interdisciplinary team of experts in this study developed the methodological tool using both grey and published literature, especially the WHO-ITU national eHealth strategy toolkits and FAIR guideline [7, 9]. The Hamilton (2013) study provided evidence of the effectiveness of the WHO-ITU eHealth strategy, while several studies published from Ethiopia, Nigeria and Uganda have critiqued policies using the FAIR Guideline [11–13]. This indicates their utility and widespread acceptability. An iterative approach was utilized to identify and define each domain and subdomain through a series of six meetings over 8 weeks to finalize the eHealth National Strategy Rating Tool (eNSRT). The study researchers read, refined and reviewed the tool before it was piloted for practical applicability on some digital health strategies of countries outside the African continent. After piloting, the tool scoring system was adjusted to improve its discriminant nature and retested again until a consensus was reached on the eNSRT. The tool included five domains which are (i) eHealth national vision and strategy, (ii) implementation plan, (iii) monitoring and evaluation component, (iv) FAIR mention and (v) recency of strategy development. See Table 1 for detailed description.

**Table 1:** The domains, subdomains and their description of the eNSRT

Domain	Subdomain	Description
Ehealth vision and strategy	Rationale for eHealth	The country’s strategic document clearly justifies the need and challenges arising that necessitated the development of the eHealth policy
	Desired Outcomes/Objectives	The strategy clearly outlines the specific objectives and outcomes for the health sector
	Foundation for Change	The policy clearly highlights the needed leadership and governance, strategy, investment, infrastructure, legislation, policy and workforce to create change
Implementation plan	Integrated Action Plan	A comprehensive action plan that states what will be done per time (year), which office or department is leading or supporting the implementation
	Implementation Phases	A clear definition and distinction of the phases of implementation of the eHealth strategy
	Action Line	Clearly states key actions or activities to be implemented/conducted for eHealth strategy
	Resource and finance mobilization - funding and	Has a budget to fund the eHealth/digital health sector’s strategic plan and/or states potential sources for mobilization of resources/states how the strategy will be funded
M & E component	Define monitoring and evaluation outcome indicators	Clearly defines indicators used to monitor and evaluate eHealth strategy
	State baseline and target measures	Has a baseline measure (what is already known) in the eHealth sector and establishes target measure (what needs to be achieved)
	Define governance and process	Clearly states who leads the eHealth strategy implementation and what processes in place for adoption at scale
Recency of strategy development		This pinpoints the recency of the years the strategy document covers. The strategy document that are recent and updated suggest that the country priorities eHealth as well as eHealth strategies in their region.
Fair mention		Whether the strategy explicitly mentions or does not that eHealth data management system of the country will follow the FAIR (Findable, Accessible, Interoperable and Reusable) guidelines in its eHealth strategy

#### Step 2: identification of relevant documents

A systematic and thorough search was conducted for publicly available national eHealth strategy documents across the official websites of ministries of health, national health authorities, online (using Google and Google Scholar), WHO websites and other relevant government agencies in African countries. The search was performed between January and March 2024. The search string included terms related to (i) Digital Health (such as ‘eHealth’, ‘Digital Health’, ‘Health Information System’) AND (ii) Policy (such as ‘Strategy’, ‘Policy,’ ‘Framework’, ‘Guidance’) AND (iii) Country (such as ‘Ghana’, ‘Nigeria’, ‘National’, among others). After a thorough search and identification of documents by all authors between December 2023 and March 2024, additional searches for national strategies that were not found were conducted in April and May. Each author was involved in the search of the strategy documents for all 54 African States to increase the chances of finding these documents which yielded some positive results. In cases where more than one national eHealth strategy documents were found, we retained and utilized the most recent and relevant version.

#### Step 3: screening and selection of relevant documents

To minimize selection bias, two independent reviewers (I.I.O. and O.P.I.) screened all the retrieved documents and selected those that met the inclusion criteria. The inclusion criteria required the documents to be official national-level eHealth policies or strategies. Documents that were published before the year 2000 or were project-specific digital health reports without national-level coverage were excluded. One eHealth national strategy document was selected and reviewed for each country.

#### Step 4: data extraction and analysis

Two independent reviewers extracted data using the code book. This included details related to the demographic details (such as the country name, strategy title and publication year) and the eNSRT, which included the five domains described above. The data were scored and analyzed.

## SCORING

### Domain scoring

For domain 1, the eHealth National Vision and Strategy was coded as ‘Complete’ if the strategy document satisfactorily described and included all the subdomains under this domain. It was scored as ‘Incomplete’ if it included one or more but not all the subdomains and scored as ‘Missing’ if it did not include any. For domains 1 to 3, domains that were marked as ‘Complete’ were scored ‘2’ points, ‘Incomplete’ were scored ‘1’ point and ‘Missing’ was awarded ‘0’. Lastly, for domain 4 (the recency of the strategy development), strategies were scored ‘2’ if ‘Updated’ (meaning the year of eHealth Strategy included 2023 or a future year), ‘1’ if ‘Present but not updated within the last 5 years (2018 to 2022)’ and ‘0’ if present but not updated before 2018 (2000 to 2018). Lastly, domain 5 (FAIR Mention) was the only domain that was dichotomized. Those who did not mention the FAIR Guideline in their strategy were scored ‘0’, while those who mentioned the FAIR Guideline were scored ‘1’ (Table 2).

**Table 2:** Scoring table for the eHealth National Strategy Tool

Domain	1. National vision and strategy	2. Implementation plan	3. Monitoring and Evaluation component	4. Recency of eHealth Strategy	5. FAIR Guideline Mention
Subdomains	a) Rationale for eHealth b) Desired Outcomes c) Foundation for Change	a) Integrated Action Plan b) Implementation Phases c) Action Line d) Resource and finance mobilization	a) Define indicators b) Define baseline and target measures c) Define governance and process		
Scoring	0. Missing	0. Missing	0. Missing	0. If present but not updated before 2018 (2000 to 2018)	0. No FAIR Guideline mention
	1. Incomplete	1. Incomplete	1. Incomplete	1. Present but not updated within the last 5 years (2018 to 2022)	1. FAIR Guideline Mention
	2. Complete	2. Complete	2. Complete	2. If present and updated till 2023	

### Overall scoring

The highest score possible after adding the highest possible score in all domains was 9 points. National eHealth policies that scored between 0 and 3 were categorized as ‘Weak’, those that scored between 4–6 were categorized as ‘Moderate’ and those that had 7–9 were categorized as ‘Strong’.

#### Step 5: data synthesis and reporting

The data were synthesized to provide a comprehensive overview of the current state of national eHealth policies and strategies in the selected African countries. The results are presented in narrative and map formats.

### Reliability and validity

For rigor, aside from ensuring good face validity by the research team and experts reviewing the eNSRT tool, an inter-rater reliability test was conducted by calculating the percent agreement in the rating between the three independent reviews on a random sample of 10 national eHealth strategies. The percent agreement was 90 (9 out of 10 strategy documents had a consistent rating), 80, 90, 100 and 100% for domains 1 (national vision and strategy), 2 (Implementation plan), 3 (Monitoring and Evaluation component), 4 (recency of eHealth strategy) and 5 (FAIR Guideline mention) respectively. The overall rating of the 10 randomly selected strategy documents was consistently rated the same by all three researchers, giving an overall rating of 100%. Based on a few differences found in the domain scores, after their independent review and rating, the three reviewers met and reached a consensus on each strategy document rating.

## RESULTS

### Overview

The national digital or eHealth strategies for 34 African countries were found. Out of these, only 16 were updated (years covered by the strategy documents covered 2023 or future years). Table 3 shows the names of the eHealth strategy, countries, year of publication and years covered by the strategy are found below.

**Table 3:** Information about the digital or eHealth strategy documents in Africa

Name of country	Name of eHealth/digital health strategy document	Year of publication	Years covered by strategy
Algeria	N/A		
Angola	N/A		
Benin	StratégieNationale de Cybersanté	2017	2018–2022
Botswana	The eHealth Strategy of Botswana	2020	2020–2024
Burkina Faso	CyberstratégiesectorielleeSanté	2016	2016–2020
Burundi	Plan National de Développement de l’Informatique de Santé du Burundi (PNDIS)	2015	2020–2024
Cabo Verde	N/A		
Cameroon	THE 2020–2024 National Digital Health Strategic Plan	2020	2020–2024
Central African Republic (CAR)	N/A		
Chad	N/A		
Comoros	Stratégie Nationale de Cybersanté 2017 to 2021	2016	2017–2021
Congo, Democratic Republic of the	Plan National de Développement de l’Informatique de la Santé (PNDIS)	2014	2020–2024
Congo, Republic of the	N/A		
Cote d’Ivoire	PLAN STRATEGIQUE DE CYBERSANTE	2011	2012–2016
Djibouti	N/A		
Egypt	National ICT Strategy 2012–2017	2012	2012–2017
Equatorial Guinea	N/A		
Eritrea	N/A		
Eswatini	eHealth Strategy 2016–2020	2016	2016–2020
Ethiopia	Information Revolution Strategic Plan 2018–2025	2018	2018–2025
Gabon	Schéma Directeur Stratégique du Système d’Information de Santé du Gabon (SDSSIS)	2017	2017–2022
Gambia	The Gambian ICT4D-2012 Plan	2008	2012-
Ghana	Policy and Strategy on Digital Health	N/A	2023–2027
Guinea	Plan National DE Developpement SANITAIRE	2015	2015–2024
Guinea-Bissau	N/A		
Kenya	Kenya National eHealth Policy 2016–2030	N/A	2016–2030
Lesotho	ICT Policy for LESOTHO	2015	2005–2015
Liberia	Liberian Health Information System & ICT Strategic Plan 2016–2021	2016	2016–2021
Libya	N/A		
Madagascar	Plan Stratégique de Renforcement du Système d’Information Sanitaire 2018–2022	2017	2018–2022
Malawi	National Digital Health Strategy 2020–2025	2020	2020–2025
Mali	N/A		
Mauritania	N/A		
Mauritius	N/A		
Morocco	N/A		
Mozambique	PLANO ESTRATÉGICO DO SISTEMA DE INFORMAÇÃO PARA A SAÚDE (SIS) 2009–2014	2009	2009–2014
Namibia	National eHealth Strategy 2021–2025	2020	2021–2025
Niger	StratégieNationale E-santé 2019–2023	2019	2019–2023
Nigeria	National Health ICT Strategic Framework 2015–2020	2015	2015–2020
Rwanda	The National Digital Health Strategic Plan 2018–2023	N/A	2018–2023
Sao Tome and Principe	N/A		
Senegal	Plan Strategique Sante Digitale 2018–2023	N/A	2018–2023
Seychelles	N/A		
Sierra Leone	National Digital Health Strategy 2018–2023	2018	2018–2023
Somalia	N/A		
South Africa	National Digital Health Strategy for South Africa 2015–2019	2015	2019–2024
South Sudan	N/A		
Sudan	Proposals for a Sudan eHealth Strategy	2015	2005-
Tanzania	THE NATIONAL DIGITAL HEALTH STRATEGY 2019–2024	2019	2019–2024
Togo	Le Plan Stratégique de Développement de la cybersanté (PSDC) 2013–2015	2012	2013–2015
Tunisia	N/A		
Uganda	Uganda National eHealth Strategy 2017–2021	N/A	2017–2021
Zambia	eHealth Strategy 2017–2021	N/A	2017–2021
Zimbabwe	Zimbabwe’s E-Health Strategy 2012–2017	N/A	2012–2017

### Domain results

For domain 1 (National Vision and Strategy), 16 policies were found to be ‘Complete’ and the other 18 were ‘Incomplete’. For domain 2 (the Implementation Plan), 9 national eHealth strategies were ‘Complete’, 24 were ‘Incomplete’ and 1 was ‘Missing’. The monitoring and evaluation component (domain 3) was ‘Complete’, ‘Incomplete’ and ‘Missing’ in 14, 16 and 4 policies, respectively. For domain 4 (recency of eHealth strategy), 16 policies were found to be recent (strategy includes the year 2023 and above), 10 policies were found to be outdated and have covered between 2018 and 2022, and 8 policies were found to be outdated and before 2018. None of the strategies mentioned or discussed extensively about the FAIR Guideline (domain 5). More information about the results per domain is found in Table 4 below.

**Table 4:** Results per domain for the African national eHealth strategies

		Domain 1	Domain 2	Domain 3	Domain 4	Domain 5		
S/N	Country	National vision and strategy	Implementation	Monitoring and evaluation	Recency of eHealth policy	FAIR mention	Overall rating	Overall rating category
1	Benin							Moderate
2	Botswana							Moderate
3	Burkina Faso							Weak
4	Burundi							Strong
5	Cameroon							Moderate
6	Comoros							Moderate
7	Congo, Democratic Republic of the							Moderate
8	Cote d’Ivoire							Weak
9	Egypt							Weak
10	Eswatini							Weak
11	Ethiopia							Moderate
12	Gabon							Moderate
13	Gambia							Weak
14	Ghana							Moderate
15	Guinea							Strong
16	Kenya							Moderate
17	Lesotho							Weak
18	Liberia							Strong
19	Madagascar							Moderate
20	Malawi							Strong
21	Mozambique							Moderate
22	Namibia							Strong
23	Niger							Strong
24	Nigeria							Moderate
25	Rwanda							Moderate
26	Senegal							Moderate
27	Sierra Leone							Strong
28	South Africa							Moderate
29	Sudan							Weak
30	Tanzania							Strong
31	Togo							Moderate
32	Uganda							Strong
33	Zambia							Moderate
34	Zimbabwe							Weak

NB: The overall ratings of the African national eHealth strategies were categorized as 'weak', 'moderate' and 'strong'.

### Overall result

**Figure 1:**
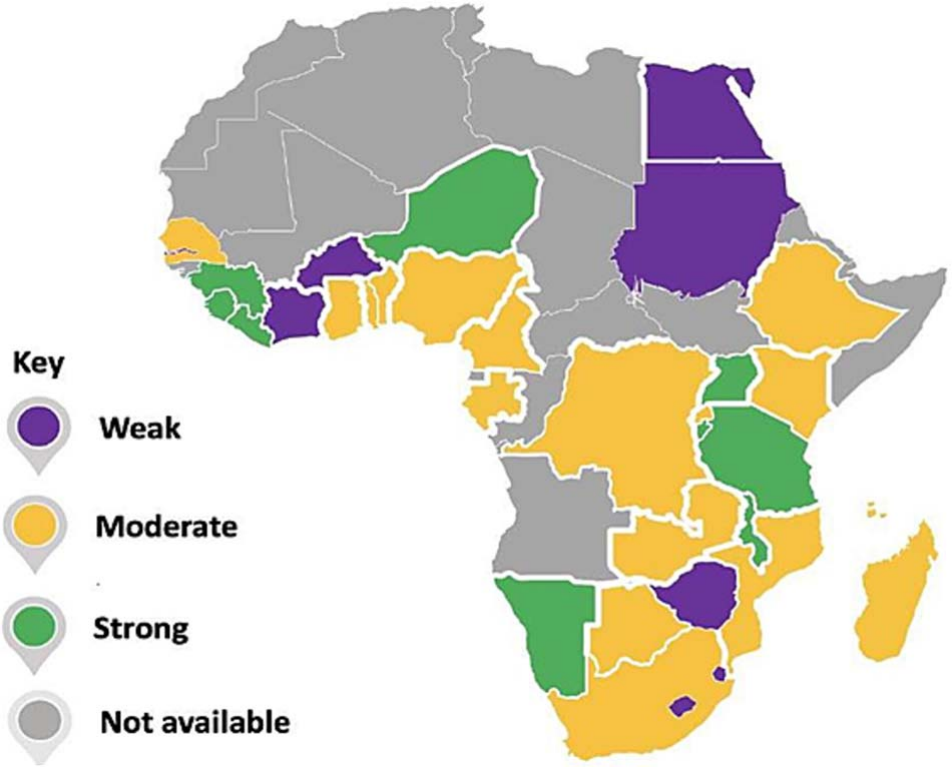
Map of the national eHealth policies in Africa.

After the scoring process, 8 national eHealth policies were ‘Weak’, 17 were ‘Moderate’ and 9 were ‘Strong’. The countries and the rating of their eHealth policies are found in the map in Fig. 1 and Table 4.

## DISCUSSION

This study provides a comprehensive review of the national eHealth policies and strategies across African countries, using internationally recognized frameworks and guidelines as the assessment criteria. As the first study to conduct such a rigorous, continent-wide analysis of digital health strategy documents in Africa, the findings reveal a mixed landscape in terms of the development, implementation and prioritization of national eHealth initiatives across the African continent.

While nine [9] countries have made significant strides in formulating robust digital health strategies, 17 countries had ‘moderate,’ and 8 countries had ‘weak’ eHealth policies. Hence, more than two-third of the policies reviewed still lack the comprehensiveness to guide the integration of digital technologies in their healthcare systems. The variability in the quality and comprehensiveness of national eHealth policies across Africa can be attributed to several factors, including limited understanding of healthcare systems and digital infrastructure, lack of standardized guidelines and resource constraints. Many countries are in the early stages of integrating digital health, and the adoption and adaptation of international frameworks like the WHO-ITU eHealth Strategy toolkit vary, leading to inconsistencies in policy development and implementation [14].

Furthermore, this study found the eHealth policies of only 34 countries to be publicly available. Countries that have not yet developed or publicly shared their eHealth strategy documents may be lagging in their efforts to harness the potential of digital technologies to improve healthcare access, quality and outcomes [15]. The lack of transparency and accessibility of these critical policy frameworks can hinder the ability of stakeholders, including policymakers, healthcare providers, digital health investors and technology companies, to align their digital health initiatives with national priorities and strategies [16].

The analysis of the national vision and strategy domain shows that 16 out of the 34 countries with available policies have a ‘complete’ articulation of the rationale, desired outcomes and foundation for change in their eHealth agenda indicating a clear strategic direction and understanding of the value proposition of digital health solutions. Those lacking in this area will need a stronger political commitment and a more coherent, evidence-based justification for their digital health priorities.

The implementation plan domain, which evaluates the comprehensiveness of action plans, resource mobilization strategies and phased implementation approaches, reveals critical gaps. Only nine countries were found to have a ‘complete’ implementation plan, while 24 had ‘incomplete’ plans. This disparity highlights the challenge many African nations face in translating their eHealth visions into tangible, well-resourced and actionable roadmaps. The monitoring and evaluation (M&E) component, another essential aspect of effective policy implementation, was also found to be lacking in many countries. Only 14 nations had a ‘complete’ M&E framework, with clearly defined indicators, baseline measures and governance structures. This deficiency limits the ability of policymakers and stakeholders to track progress, measure the impact of digital health interventions and make data-driven decisions to optimize program delivery.

Notably, the study found that only 16 out of the 34 countries had updated their eHealth policies to include the year 2023 or beyond, indicating a lack of consistent strategy review and renewal processes in many African nations. This finding suggests that the digital health landscape in Africa may not be keeping pace with the rapid advancements in technology and the evolving healthcare needs of the population. Furthermore, the analysis revealed a surprising absence of explicit mentions or alignment with the FAIR (Findable, Accessible, Interoperable and Reusable) data principles in any of the reviewed documents. This oversight is concerning, as the FAIR Guidelines play a crucial role in ensuring the effective management and stewardship of health data, a critical enabler of digital health innovation and evidence-based decision-making [9]. Mamuye and other scholars have also emphasized the importance of interoperability and health information exchange inclusion in policies [17, 18].

The insights gained from this study have important implications for policymakers, healthcare stakeholders and international development partners working to strengthen digital health ecosystems in Africa. The findings highlight the need for a renewed focus on the development, implementation and evaluation of national eHealth strategies that are coherent, well-resourced and aligned with global best practices such as the WHO-ITU national eHealth strategy toolkit, FAIR guidelines and the Global Strategy on Digital Health 2020–2025 [6, 7, 9]. Furthermore, ensuring that these critical strategy documents are publicly available and easily accessible can enable healthcare providers, technology companies, civil society organizations and the general public to contribute to the policymaking process and hold governments accountable for the implementation and outcomes of their digital health agendas to improve healthcare access, quality and outcomes for their populations.

### Strengths of this study

This study is the first to perform a comprehensive review of a wide range of publicly available national-level eHealth strategy documents from 34 African countries, providing relevant insight to inform both regional and continental-wide strategies in Africa. Also, the rigorous methodology adhered to the well-established Arksey and O’Malley scoping review approach, ensuring a systematic and transparent process for the identification, selection and analysis of relevant strategy documents. The evaluation of the national eHealth strategies utilized the eNSRT which built upon evidence-based international guidelines (the WHO-ITU national eHealth strategy toolkit and the FAIR guidelines), aligning the study with globally recognized best practices and standards for eHealth policy development.

The study’s focus on the African continent is particularly relevant, as the region has witnessed a surge in digital health initiatives in recent years, accompanied by the need for robust policy frameworks to guide their effective integration and scale-up within healthcare systems. The study’s findings culminate in a series of practical recommendations to support African countries in strengthening their national eHealth strategy development and contributing to the actualization of the African Union Agenda 2063.

### Limitations of the study

One of the key limitations of this study is the heavy reliance on publicly available national eHealth strategy documents. Some countries may have developed and implemented eHealth policies that are not publicly accessible. Furthermore, the study did not involve direct engagement with policymakers or other stakeholders, which could have provided additional insights. The study’s scope was limited to the analysis of strategy documents and did not examine the actual implementation and impact of the national eHealth strategies on the ground. Future research should delve deeper into the contextual factors, political dynamics and resource constraints that have influenced the formulation and implementation of national eHealth strategies and track their impact on healthcare delivery and population health outcomes.

## CONCLUSION

The findings of this comprehensive review highlight the uneven progress in the development and implementation of national eHealth policies across the African continent. While some countries have made significant strides in establishing robust policy frameworks to guide the integration of digital health solutions, many others are still lagging. Addressing the gaps identified in this study will be crucial for African nations to harness the transformative potential of digital technologies and ensure equitable access to quality healthcare for their populations. It will further contribute to the growth and development of Africa’s health sector, which in turn will allow for the development of Africa’s human and technological resources. As digital health continues to evolve, the insights generated by this study can inform policymakers, healthcare providers, researchers, investors, public health professionals and technology companies in their efforts to align national strategies with global best practices and the unique needs of African healthcare systems.

## STUDY FUNDING AND APC FUNDING

No funding was received.

## Data Availability

The datasets that supports the findings of this study were derived from sources in the publicly available national eHealth strategy document from African nations, which were obtained through a systematic search. These eHealth strategy documents were published between 2000 and 2023 and are available in public domain.

## References

[1] Dodoo JE, Al-Samarraie H, Alsswey A. The development of telemedicine programs in sub-Saharan Africa: progress and associated challenges. *Heal Technol* 2022;12:33–46. 10.1007/s12553-021-00626-7 PMC8613515 34849325

[2] Stoumpos AI, Kitsios F, Talias MA. Digital transformation in healthcare: technology acceptance and its applications. *Int J Environ Res Public Health* 2023;20:3407. 10.3390/ijerph20043407 36834105 PMC9963556

[3] Mbunge E, Batani J. Application of deep learning and machine learning models to improve healthcare in sub-Saharan Africa: emerging opportunities, trends and implications. *Telemat Inform Rep* 2023;11:100097–100107. 10.1016/j.teler.2023.100097

[4] Owoyemi A, Osuchukwu JI, Azubuike C et al. Digital solutions for community and primary health workers: lessons from implementations in Africa. *Front Digit Health* 2022;4:876957. 10.3389/fdgth.2022.876957 35754461 PMC9215204

[5] Jandoo T. WHO guidance for digital health: what it means for researchers. *Digit Health* 2020;6:2055207619898984. 10.1177/2055207619898984 31949918 PMC6952850

[6] WHO. Global strategy on digital health 2020–2025. Geneva: World Health Organization, 2021. https://www.who.int/publications/i/item/9789240020924 (21 June 2024, date last accessed).

[7] WHO-ITU. WHO-ITU national eHealth strategy toolkit. Steering committee: Najeeb Al-Shorbaji, Joan Dzenowagis (WHO); Mario Maniewicz, Hani Eskandar (ITU). 2012. https://www.itu.int/ITU-D/cyb/events/2012/e-health/Nat_eH_Dev/Session%201/ITU-WHO%20National%20eHealth%20Strategy%20toolkit%20March%202012.pdf

[8] Hamilton C. The WHO-ITU national eHealth strategy toolkit as an effective approach to national strategy development and implementation. *MEDINFO* 2013;192:913–6. 23920691

[9] Wilkinson MD, Dumontier M, Aalbersberg IJ et al. The FAIR guiding principles for scientific data management and stewardship. *Scientif data* 2016;3:1–9. 10.1038/sdata.2016.18 PMC4792175 26978244

[10] Inau ET, Nalugala R, Nandwa WM et al. FAIR equivalency, regulatory framework and adoption potential of FAIR guidelines in health in Kenya. *Data Intell* 2022;4:852–66. 10.1162/dint_a_00175

[11] Kawu AA, Elijah J, Abdullahi I et al. FAIR guidelines and data regulatory framework for digital health in Nigeria. *Data Intell* 2022;4:839–51. 10.1162/dint_a_00174

[12] Basajja M, Van Reisen M, Oladipo F. FAIR equivalency with regulatory framework for digital health in Uganda. *Data Intell* 2022;4:771–97. 10.1162/dint_a_00170

[13] Tadele Taye G, Yohannes Amare S, Gebremeskel GT et al. FAIR equivalency with regulatory framework for digital health in Ethiopia. *Data Intell* 2022;4:813–26. 10.1162/dint_a_00172

[14] Global Digital Health Monitor. State of digital health. 2023 https://digitalhealthindex.squarespace.com/s/State-of-Digital-Health_2023.pdf (21 June 2024, date last accessed).

[15] WHO. Global observatory for eHealth policies. 2023. https://www.who.int/observatories/global-observatory-for-ehealth (21 June 2024, date last accessed).

[16] Gagliardi AR, Brouwers MC. Lack of transparency in health policy frameworks can impede stakeholder alignment. *Health Res Policy Syst* 2020;18:90. 10.1186/s12961-020-00609-432787858

[17] Mamuye AL, Yilma TM, Abdulwahab A et al. Health information exchange policy and standards for digital health systems in africa: a systematic review. *PLOS Digit Health* 2022;1:e0000118. 10.1371/journal.pdig.0000118 36812615 PMC9931258

[18] Olu O, Muneene D, Bataringaya JE et al. How can digital health technologies contribute to sustainable attainment of universal health coverage in Africa? A perspective. *Front Public Health* 2019;7:341. 10.3389/fpubh.2019.00341 31803706 PMC6873775

